# Profiles of muscle-specific oxygenation responses and thresholds during graded cycling incremental test

**DOI:** 10.1007/s00421-024-05593-1

**Published:** 2024-09-11

**Authors:** Carlos Sendra-Pérez, Alberto Encarnacion-Martinez, Rosario Salvador-Palmer, Juan M. Murias, Jose I. Priego-Quesada

**Affiliations:** 1 Research Group in Sports Biomechanics (GIBD), Department of Physical Education and Sports, Faculty of Physical Activity and Sport Sciences, Universitat de València, St: Gascó Oliag, 3. 46010, Valencia, Spain; 2 Red Española de Investigación del Rendimiento Deportivo en Ciclismo y Mujer (REDICYM), Universitat de València, Ontinyent, Spain; 3 Biophysics and Medical Physics Group, Department of Physiology, Universitat de València, Valencia, Spain; 4 College of Health and Life Sciences, Hamad Bin Khalifa University, Doha, Qatar

**Keywords:** Muscle oxygenation, Breakpoint, Exercise, Cycling

## Abstract

Compared to the determination of exercise thresholds based on systemic changes in blood lactate concentrations or gas exchange data, the determination of breakpoints based on muscle oxygen saturation offers a valid alternative to provide specific information on muscle-derived thresholds. Our study explored the profiles and timing of the second muscle oxygenation threshold (MOT2) in different muscles. Twenty-six cyclists and triathletes (15 male: age = 23 ± 7 years, height = 178 ± 5 cm, body mass = 70.2 ± 5.3 kg; 11 female: age = 22 ± 4 years, height = 164 ± 4 cm, body mass = 58.3 ± 8.1 kg) performed a graded exercise test (GXT), on a cycle ergometer. Power output, blood lactate concentration, heart rate, rating of perceived exertion, skinfolds and muscle oxygen saturation were registered in five muscles (vastus lateralis, biceps femoris, gastrocnemius medialis, tibialis anterior and triceps brachii) and percentage at which MOT2 occurred for each muscle was determinated using the Exponential Dmax. The results of Statistical Parametric Mapping and ANOVA showed that, although muscle oxygenation displayed different profiles in each muscle during a GXT, MOT2 occurred at a similar percentage of the GXT in each muscle (77% biceps femoris, 75% tibalis anterior, 76% gastrocnemius medialis and 72% vastus lateralis) and it was similar that systemic threshold (73% of the GXT). In conclusion, this study showed different profiles of muscle oxygen saturation in different muscles, but without notable differences in the timing for MOT2 and concordance with systemic threshold. Finally, we suggest the analysis of the whole signal and not to simplify it to a breakpoint.

## Introduction

Within the past two decades, near-infrared spectroscopy (NIRS) technology has been extensively used to investigate local muscle oxygenation responses during exercise (Caen et al. 2022; Murias et al. 2013; Perrey et al. 2024). Recently, the popularity of NIRS technology has grown in sports science due to the availability of low-cost devices, as well as user-friendly wearables that have enabled more refined analysis of skeletal muscle oxygenation (Perrey et al. 2024). NIRS has been used in sports sciences for assessing acute local muscle changes (Boone et al. 2016; Craig et al. 2017), or the acute and chronic effect of an exercise training program on muscle oxygenation profiles (Jones et al. 2018; Wang et al. 2023), among others. However, one of the most researched and widely used applications, is the determination of muscle oxygenation breakpoints during exercise testing (Batterson et al. 2023; Caen et al. 2022; Iannetta et al. 2017a, b; Rodrigo-Carranza et al. 2021; Salas-Montoro et al. 2022), some of which have been associated to metabolic boundaries (i.e., thresholds) separating exercise intensity domains (Bellotti et al. 2013; Feldmann et al. 2022; Keir et al. 2015a; Sendra-Pérez et al. 2023b).

When compared with the determination of exercise thresholds based on the systemic changes in blood lactate concentrations or gas exchange data to interpret changes/disruptions to metabolic homeostasis within the exercising muscles, the determination of breakpoints by NIRS allows for a more specific evaluation of the changes taking place within active muscles (Perrey & Ferrari 2018). For example, the second muscle oxygenation threshold (MOT2) of the major muscle involved in power production during cycling exercise (e.g., vastus lateralis), has been shown to occur at a similar intensity as to the respiratory compensation point (RCP) (Rodrigo-Carranza et al. 2021), and the second lactate threshold (LT2) (Salas-Montoro et al. 2022). Importantly, beyond some case studies (Paulauskas et al. 2022; Stöggl & Born 2021), most of the studies examining NIRS breakpoints have only assessed one muscle, such as the vastus lateralis (Cayot et al. 2021), rectus femoris (Salas-Montoro et al. 2022), deltoideus (Yogev et al. 2022), gastrocnemius (Borges & Driller 2016), or intercostals (Contreras-Briceño et al. 2022).

Thus, whereas several studies have assessed MOTs in one muscle, there is a scarcity of studies synchronously evaluating NIRS-derived MOTs from different muscles during exercise (Batterson et al. 2023; Iannetta et al. 2017b; Yogev et al. 2022). Moreover, there is also a lack of scientific information about whether the MOTs of different muscles occur at different times, and if some are more related to the systemic threshold (e.g., LT2) than others. Then, it is necessary to further evaluate muscle oxygen saturation (SmO_2_) profiles and their derived MOTs during exercise, as MOTs might not be uniform in all muscles during cycling, as reported by Batterson et al. (2023) in running. Thus, exploring the overall profiles and occurrence of NIRS-derived thresholds on different muscles will contribute to better understand the importance of applying NIRS devices in multiple locations during exercise testing.

The present study aimed to explore the profiles of muscle-specific oxygenation responses and timing of MOT2 in relation to power output from different muscles including: one power-generating muscle (vastus lateralis); three stabilizing muscles (gastrocnemius medialis, tibialis anterior, and biceps femoris) (Park & Caldwell 2021); and one control muscle (triceps brachii) during a graded cycling exercise test (GXT) to task failure. We hypothesized that i) the power output generating muscle would have a greater O_2_ extraction earlier during the GXT in line with their profile of recruitment; ii) the estimation of timing at which the MOT2 occurred would not differ between the three stabilizing muscles and power-generating muscle.

## Materials and methods

### Participants and ethical approval

The research involved a sample of 26 cyclists and triathletes (15 males: age = 23 ± 7 years, height = 178 ± 5 cm, body mass = 70.2 ± 5.3 kg; 11 females: age = 22 ± 4 years, height = 164 ± 4 cm, body mass = 58.3 ± 8.1 kg). Participants were triathletes and cyclists who trained 12 ± 3 h per week, with a cycling experience of 9 ± 5 years, and who were categorized as competitive (Priego Quesada et al. 2018). The inclusion criteria included to be nonsmokers, between 18 and 40 years of age, with at least six months of experience in cycling or triathlon, and with at least two cycling workouts per week. The study protocol was reviewed and approved by the Ethics Committee at the University of Valencia (No. 2630853) and followed the ethical principles for medical research in humans established in the Declaration of Helsinki.

### Experimental protocol

Participants were instructed to sleep for a minimum of seven hours on the night before the test and were advised not to consume caffeine and to avoid performing exercise within 24 h prior to GXT. The protocol was performed on a cycle ergometer (Cardgirus W3, Sabadell, Spain), which was previously adjusted to each cyclist’s bike measurements. The GXT started with a 3-min constant load of 1 W·kg^−1^ during the warm-up, followed by 3-min steps each with power output increments of 0.5 W·kg^−1^ (Possamai et al. 2020). Participants were asked to maintain the cadence around 90 ± 10 rpm during GXT. Task failure was considered when the cadence was below 80 rpm for more than 10-s or when the participant decided to give up despite strong verbal encouragement. For consistency, eumenorrheic females were tested during the early days of the follicular phase, and females who used a contraceptive pill performed the test during the abstinence week. Nevertheless, it has been shown that the phase of the menstrual cycle has no significant effects on maximal and submaximal performance outcomes (Mattu et al. 2020).

### Procedures and data analysis

Power output outcomes during testing were normalized to body mass (W·kg^−1^). Blood lactate concentration (mmol·L^−1^) obtained from earlobe blood samples in the last 30-s of each step was measured using a Lactate Pro 2 analyzer (AKRAY Europe BV, Amstelveen, Netherlands). The power output at which the second lactate threshold (LT2) occurred was estimated by using RStudio “lactater” library (version 2022.02.03), using Exponential Dmax method, and the percentage of the GXT where the LT2 occured.

The SmO_2_ (%) was monitored continuously during the entire GXT and it was assessed using NIRS technology at a sampling frequency of 0.5 Hz (Moxy Monitor, Fortiori Design LLC, Minneapolis, USA). The Moxy detectors are spaced at 12.5 mm and 25 mm from the emitter. Moxy devices were fixed using Hipafix™ in five locations on the left or right side of the body depending on the dominant side, which was determined by asking the question “If you would shoot a ball on a target, which leg would you use to shoot the ball?” (Melick et al. 2017). The locations of NIRS probes placement included: i) vastus lateralis muscle at 2/3 of the distance between the line from the anterior superior iliac spine to the lateral side of the patella; ii) the long head of the biceps femoris muscle at 1/2 of the distance between the line between the ischial tuberosity and the lateral epicondyle of the tibia; iii) gastrocnemius medialis at the most prominent bulge of the muscle; iv) tibialis anterior at 1/3 of the distance between the tip of the fibula and the tip of the medial malleolus; and v) triceps brachii at 1/2 of the distance between the long head on the line between the posterior crista of the acromion and the olecranon at two finger width medial to the line. Placement of devices was based on the surface electromyography guidelines (Hermens et al. 1999). The SmO_2_ data were filtered using a Butterworth low-pass filter (cutoff frequency of 0.2 Hz). To determine where occurred the MOT2 the following approach was employed. Firstly, the variation of the SmO_2_ (∆SmO_2_) was calculated as the difference between the average value from the warm-up step and the rest of the GXT (Sendra-Pérez et al. 2023a, b). Secondly, the ∆SmO_2_ data were inverted behaved incrementally (i.e., like lactate) (Sendra-Pérez et al. 2023a, b). Finally, MOT2 for each muscle was determinated using the Exponential Dmax method, as this method showed it to offer the best fit (Sendra-Pérez et al. 2023a, b).

In addition, the percentage of the GXT at which LT2 or MOT2 occurred was calculated considering the steps completed during the GXT.

## Statistical analysis

Statistical analysis was performed using RStudio (version 2022.02.03) and Python (Annaconda Navigator 2.3.2). As all of the variables followed a normal distribution (p > 0.05; Shapiro–Wilk test), mean and standard deviation were used to present the data. The significance level was set at p < 0.05. To compare the ∆SmO_2_ responses during the entire GXT, one-dimensional statistical parametric mapping (SPM) techniques were used to analyze a time-series signal (∆SmO_2_) along the GXT (Pataky 2010), with 0% being the beginning of the GXT, and 100% being its end. The SPM1D package (www.spm1d.org) was used by applying an one-way repeated-measures ANOVA to analyse the differences between muscles. When significant muscle-by-muscle interactions were presented, post hoc analysis using Student’s t test with Bonferroni correction compared the differences between profiles of the ∆SmO_2_. Further, differences in the timing between percentage of the GXT at which the MOT2 in muscles involved in pedalling (i.e., vastus lateralis, gastrocnemius medialis, tibialis anterior and biceps femoris) and the systemic threshold occurred were assessed by an ANOVA. Then, the agreement between the timing of all thresholds was calculated by intraclass correlation coefficients (ICC), based on a single rater-measurement, absolute-agreement and 2-way random-effects model, and classified as follow: 1.00–0.81 (excellent), 0.80–0.61 (very good), 0.60–0.41 (good), 0.40–0.21 (reasonable) and 0.20–0.00 (deficient) (Weir 2005). Finally, Bland–Altman plots and limits of agreement (± 1.96 SD) were performed for the percentage of the GXT at which MOT2 and LT2 occur to examine the agreement between MOT2 and systemic thresholds.

## Results

### Evolution of SmO_2_ during the graded cycling test

**Fig. 1:**
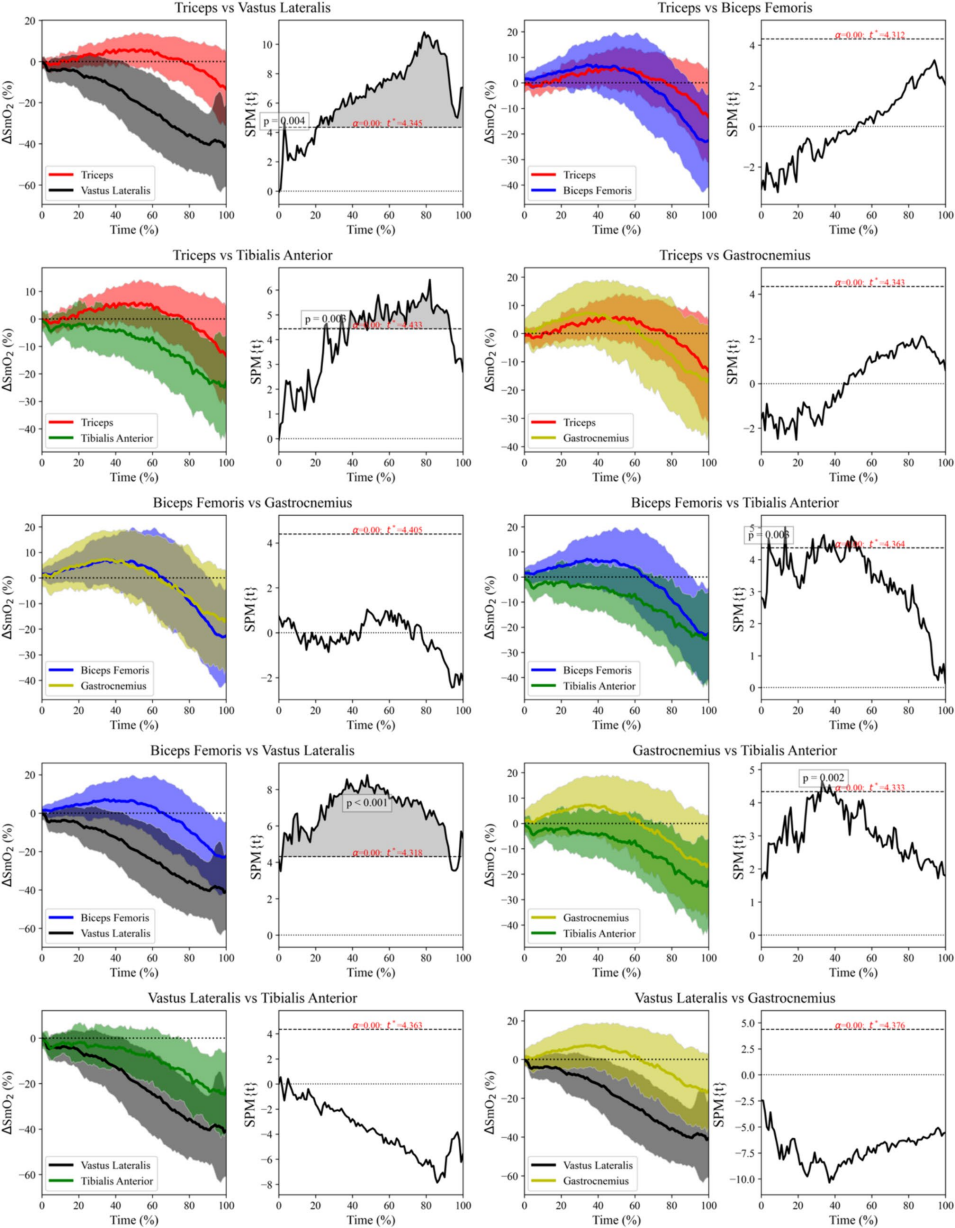
Left: Averaged time series of muscle oxygen saturation in five muscles during the graded cycling exercise test. The shaded area represent the standard deviation of the muscle oxygen saturation variation (∆SmO_2_). Right: The paired *t* test (spm1d) of muscle oxygen saturation activity of the control condition compared to the muscle oxygen saturation. The vertical axis displays the one SPM {*t*}. A significant effect is present at instances where the black line is above the upper horizontal dotted line

The SPM analyses (Fig. 1) identified different patterns of muscle oxygenation (i.e., ∆SmO_2_): the vastus lateralis consistently decreased its ∆SmO_2_ throughout the GXT; the tibialis anterior displayed a stable ∆SmO_2_ until ~ 30% and then decreased until the end of the GXT; the biceps femoris, gastrocnemius medialis and triceps brachii increased their ∆SmO_2_ until ~ 50% of the GXT, and then they showed a progressive decrease in ∆SmO_2_ and crossed the 0% line at ~ 60–80% of the GXT. The triceps brachii showed greater ∆SmO_2_ values and different timing compared to the power-producing muscle (i.e., vastus lateralis; *p* = 0.004) and the tibialis anterior (*p* = 0.001) during the entire GXT. The vastus lateralis also showed different timing, since the vastus lateralis displayed greater desaturation than the biceps femoris (*p* < 0.001). Futher, different muscles within the leg showed some differences in the first part of the GXT, as the gastrocnemius medialis had an increase in ∆SmO_2_ that was not observed in the tibialis anterior (p = 0.002). Nevertheless, the biceps femoris also presented greater ∆SmO_2_ values in the first steps (p = 0.003 vs. tibialis anterior).

### MOT2 in muscles involved in pedalling

**Fig. 2:**
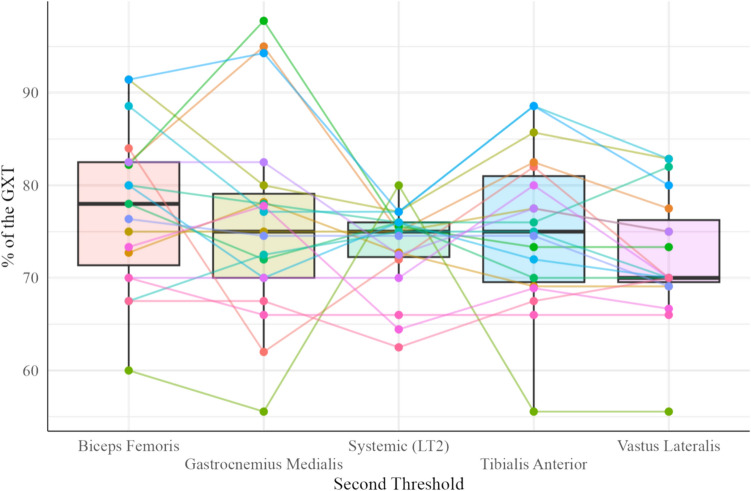
Box plot of the different percentage at which the second muscle oxygenation thresholds (biceps femoris, gastrocnemius medialis, tibialis anterior and vastus lateralis) and the systemic threshold (i.e., second lactate threshold) occurred during the graded cycling exercise test (GXT)

No differences were observed with the percentage of the GXT at which occur the MOT2 between muscles involved in pedalling and the systematic threshold (*p* = 0.07; 95% CI [0.00, 1.00] from the ANOVA) (Fig. 2). Due to the linear behavior of ∆SmO_2_ in the gastrocnemius medialis in seven participants (5 females and 2 males) which did not allow to obtain automatically the MOT2, the analysis was performed in 19 cyclists (13 males and 6 females: age = 22 ± 6 years, height = 174 ± 9 cm, and body mass = 65.6 ± 8.6 kg).

In addition, the intraclass correlation coefficients (ICC) were significant when all thresholds were compared (i.e., LT2 and MOT2) (*p* < 0.001) with a good ICC (0.51), and when only MOT2s were compared (*p* < 0.001) also showed a very good ICC (0.64) was also observed (Table 1).

### Bland–Altman plots

**Fig. 3:**
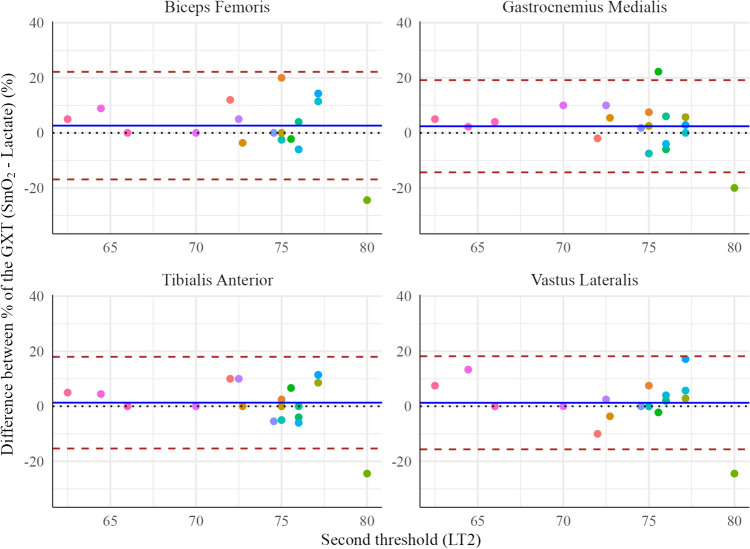
Bland–Altman plots analysis for showing the differences in the percentage of the graded cycling exercise test (GXT) at which the MOT2 in different muscles and the second threshold (LT2) occurred. The different colours shades in the dots show different participants. The central continuous blue line represents the absolute average difference between instruments (Bias), and limits of agreement red lines represent ± 1.96 standard deviations

Bland**–**Altman plots (Fig. 3) show the adjusted bias for the timing of the four muscles regarding the LT2 in the percentage of the GXT at which they occur. For the stabilizing muscles, the Bland–Altman analysis showed that the bias of biceps femoris was 2.64 ± 9.97% (CI 95% [−2.17, 7.44%], *p* > 0.05), for the gastrocnemius was 2.41±8.54% (CI 95% [−1.71, 6.53%], *p* > 0.05), and for the tibialis anterior was 1.32 ± 8.50% (CI 95% [−2.78, 5.42%], *p* > 0.05). For the power-generating muscle (i.e., vastus lateralis) a bias of 1.28 ± 8.63% (CI 95% [−2.88, 5.44%], *p* > 0.05) was found between the percentage of GXT at which MOT2 occurred and the LT2 Table 1.

**Table 1:** Reliability was calculated through the Intraclass Correlation Coefficient (ICC) at the percentage of the graded cycling exercise test (GXT) at which the second muscle oxygenation threshold and the second lactate threshold occured. Significant ICC values (*p* < 0.05) are shown in bold letters

Thresholds (% of the GXT)	ICC	CI (95%)	*p* value
Biceps femoris, gastrocnemius medialis, tibialis anterior, vastus lateralis and systemic threshold	0.51	0.31 – 0.73	< 0.001
Biceps femoris, gastrocnemius medialis, tibialis anterior and vastus lateralis	0.64	0.43 – 0.80	< 0.001

## Discussion

The purpose of the present study was to explore the differences in the timing of MOT2 between muscles involved in pedaling during GXT testing. The novelty of our study was to measure MOT2 in different muscles and to evaluate the timing (i.e., % of the GXT at which the breakpoint occurred) between the muscle-specific and systemic threshold (LT2). The main finding of this study was that, despite the different profiles in the SmO_2_ signal observed in the muscles assessed during the GXT, the MOT2 occurred at a similar percentage of the GXT (i.e., between 72 and 77% of the GXT). In addition, these were similar to the systemic threshold (73% of the GXT), displayed a good intraclass correlation coefficient, and the bias between MOT2 and LT2 in the percentage of the GXT was reduced (i.e., 2.64 ± 9.97% for stabilizing muscles, and 1.28 ± 8.63% for power-generating muscle), and not significantly for any muscle.

When considering the different profiles of neuromuscular activation from different muscles during cycling as observed in the previous studies (i.e., with the vastus lateralis showing a continuous increase of activation during an incremental test (Bini et al. 2018), and the stabilizer muscles showing a more stable activation (Hug & Dorel 2009)), different patterns of muscle deoxygenation for each muscle were expected. Our study showed that the power output generating muscle (vastus lateralis) started to deoxygenate early during the test (~ 20% of the GXT), with this profile continuing steadily until the end of the test, as observed in the study by Iannetta et al. (2017a, b). On the other hand, the gastrocnemius medialis and biceps femoris both showed reoxygenation from 0% to ~ 50% of the GXT, followed by a deoxygenation pattern, and the tibialis anterior showed a plateau during the first part of the test (~ 30%), followed by a progressive decline in the signal. This might be explained by the fact that the tibialis anterior is a small muscle that might start to fatigue earlier than other larger stabilizing muscles. Given that other studies have shown inter-muscle differences in the profiles of neuromuscular activation (Hug & Dorel 2009), the differences between muscles in the SmO_2_ response might be explained by neuromuscular activation profiles, in addition to other factors as differences in blood flow distribution and a smaller oxygen diffusing area in muscles (Calbet et al. 2005; Ozyener et al. 2012). Additionally, our study included a control muscle (triceps brachii) that was not directly involved in pedaling that showed a reoxygenation profile during the first steps, followed by a decrease that started at ~ 60% of the GXT. This is related with Yogev et al. (2022), who showed that the lateral deltoid (i.e., nonlocomotor or control muscle) presented an oxygenation more constant response during the test up to ~ 70% of the incremental cycling ramp than the vastus lateralis (locomotor or power-generating muscle), with a decrease of the ∆SmO_2_ in the lateral deltoid thereafter. However, this difference might be that the triceps brachii participates in maintaining the trunk position on the bicycle, which increases its activity as the test progresses. The oxygenation profiles of these nonlocomotor muscles could occur due to a systemic blood flow redistribution that might increase local perfusion even in tissues that are not yet displaying an increased metabolic demand (Ozyener et al. 2012).

The MOT2 was also assessed in our study. Interestingly, the MOT2 was detected at very similar percentages of the GXT, ranging from 77 to 72% of the GXT with very good ICC (0.64), and at a similar percentage of the systemic threshold (73%). These results are in agreement with Batterson et al. (2023), who showed that MOT2 from three different muscles (vastus lateralis, gastrocnemius, and biceps femoris) and LT2 occurred at similar times. Therefore, the fact that despite the different profiles of ∆SmO_2_, they all displayed the MOT2 at a similar percentage of the GXT, might indicate a similar mechanistic basis for the occurrence of these events, as it has also been proposed under different experimental conditions (Iannetta et al. 2017b; Keir et al. 2015b). Importantly, some other studies have detected the MOT2 at higher percentages of the test peak responses (Iannetta et al. 2017b; Racinais et al. 2014). This may be related to the type and characteristing of the incremental test (i.e., slow vs. fast step or ramp protocols) (Caen et al. 2021; Iannetta et al. 2019), the method for determining the MOT, or the population evaluated (Jamnick et al. 2020; Van Der Zwaard et al. 2016). Even though NIRS-derived MOTs have been shown to be a good estimation of the boundaries between exercise intensity domains (Keir et al. 2015b; Sendra-Pérez et al. 2023b), some have challenged the ability of the local thresholds to predict traditionally accepted indicators of the separation between exercise intensity domains (Caen et al. 2022). In connection to this, Caen et al. (2022) suggested that local thresholds (using NIRS or EMG) were less reliable compared to whole-body thresholds, which could be related to factors such as the adipose tissue thickness (Craig et al. 2017) and/or redistribution of blood flow to the skin that might play a role in the identification of the MOTs (Tew et al. 2010).

The MOT2 of the different muscles showed certain agreement with the LT2, with a mean bias of 3% for the stabilizing muscles and 1% for the power-generating muscle. From the study by Boone et al. (2016), many studies have been conducted to improve the determination of systemic thresholds (i.e., RCP or LT2) with noninvasive devices using NIRS technology or surface electromyography that allows the detection of local thresholds (Caen et al. 2022). A systemic review about MOTs in NIRS technology showed moderate to good reliability with LT2 (ICC = 0.80) in different muscles (e.g., vastus lateralis, rectus femoris, biceps femoris and gastrocnemius medialis) and sports (running, cycling and rowing) (Sendra-Pérez et al. 2023b). In addition, a study has recently found that the thresholds calculated with the heart rate variability and NIRS technology showed small biases and strongly agreed with the RCP calculated with the heart rate and VO_2_ (Fleitas-Paniagua et al. 2024).

Some limitations could be considered in the present study. A methodological factor to consider in the present study is related to the step size of the protocol (0.5 W·kg^−1^), as this amplitude could decrease the precision for identifying LT1 and LT2. Additionally, the MOT2 was calculated using the Exp-Dmax method, which is a mathematical method for determining an inflection point, that, although valid and commonly used (Jamnick et al. 2020), cannot be considered as a gold-standard approach and could present differences with other determination methods. Finally, the locations where the NIRS probes were located might have certain variations in the skinfold, which could also affect the results. Nonetheless, future research should explore the underlying mechanisms causing regional variations in the profile of muscle oxygenation and MOT, and if these thresholds (i.e., MOT2) respond to factors that modify threshold intensity, such as anemia, altitude, or glycogen depletion. In addition, future research should calculate how changes in blood volume within different muscles might affect the responses in order to improve the interpretation of the results.

In conclusion, this study showed that there are different profiles of muscle oxygen saturation (O_2_ extraction) depending on the muscle function during the pedaling (power output generators and stabilizers), but without notable differences between them in the determination of MOT2. Importantly, although MOT2 can be determined during cycling from different muscles, the analysis of the SmO_2_ for each muscle depicts marked differences in profile, depending on the intensity of exercise. Then, analysis of the whole signal instead of the MOT2 can add value to the interpretation of the results.

## Data Availability

The data that support the findings of this study are available from the corresponding author, [A.E.M.], upon reasonable request.
